# Referral patterns to a Middle Eastern oral medicine service: a retrospective analysis

**DOI:** 10.1186/s12913-026-14954-9

**Published:** 2026-06-13

**Authors:** Abdullah Alsoghier, Abdulaziz Aloshaywi, Salman Alnadhari, Mohammad Alrefeai

**Affiliations:** 1 Department of Oral Medicine and Diagnostic Sciences, College of Dentistry, King Saud University, Riyadh, 12372 Saudi Arabia; 2 Dental Internship Training Program, College of Dentistry, King Saud University, Riyadh, Saudi Arabia; 3 Department of Restorative Dental Science, College of Dentistry, King Saud University, Riyadh, Saudi Arabia

**Keywords:** Oral medicine, Mouth disease, Referral and consultation, Orofacial pain, Mouth ulcers, Oral diagnosis

## Abstract

**Background:**

Individuals with oral mucosal changes, oral dryness, and orofacial pain often visit multiple healthcare providers before receiving clinical care from oral medicine specialists. Such diagnostic delays may impact their oral health-related quality of life and potentially affect disease outcomes. The present study aimed to describe the patterns of referrals to an oral medicine service.

**Methodology:**

A retrospective analysis of all referrals to the Oral Medicine Clinic at King Saud University in Riyadh, Saudi Arabia, over an 8-year period (2015 to 2023), was performed. Characteristics related to referrals (referrer speciality) and patients (demographics, medical history, reason/s for referral, and involved anatomical sites) were assessed. Data analysis and visualisation were performed using Microsoft Excel [v2.92.1]. The study variables were summarised using descriptive statistics: frequencies and percentages (%) for categorical variables, and mean (± SD) and median for numerical variables.

**Results:**

Of the 500 assessed referrals, 350 were included in the analysis. The primary referral sources were general dentists and dental emergency clinics (46%). In contrast, those received from medical practitioners were as low as 8 out of the 350 referrals. Most of these referrals were for females (62%) and presented with an average age of 36 (± 16). Reasons for referral often included pain (50%) and temporomandibular joint-related complaints (44%).

**Conclusions:**

The present study described the pattern of referrals to a Middle Eastern university-based clinical service, which did not necessarily mirror international studies. Future work may consider unifying the speciality’s scope, considering the differences across global healthcare systems and their policies. There is also a need to develop clinical practice guidelines for referral pathways to ensure value-based clinical care for oral and maxillofacial diseases.

## Background

Patients are referred to oral medicine (OM) for several reasons, primarily for oral mucosal lesions or ulcerations of immune-related, neoplastic, and infectious etiologies, as well as oral manifestations of systemic diseases, temporomandibular joint disorders, orofacial pain, and salivary gland-related disorders [1]. Furthermore, dental management of medically complex patients is within the scope of OM practices worldwide [1]. As a tertiary care speciality that is often under-recognised among healthcare professionals [2], integrating the OM into healthcare systems is essential, as these conditions are often late diagnosed, affecting an individual’s quality of life and causing a significant socioeconomic burden [3, 4].

Along with the dental-related causes of pain and dysfunction, OM specialists can provide care in collaboration with other healthcare professionals to manage oral and dental health considerations for patients with various systemic diseases [1, 5]. These are often conducted in liaison with cardiologists, dermatologists, nutritionists, endocrinologists, geriatricians, geneticists, haematologists, internists, rheumatologists, neurologists, obstetrician-gynaecologists, oncologists, ophthalmologists, paediatricians, pain specialists, and psychologists [3]. Furthermore, the inevitable oral and maxillofacial complications of medical therapy (e.g., cancer-related oral mucositis, xerostomia, and osteonecrosis of the jaw) emphasise the importance of such integration to reduce diagnostic and management delays and contribute to favourable patient outcomes and experiences [5].

Despite being a recognised speciality, little is known about OM practices across different parts of the Middle East [3, 6]. Additionally, these practices may vary across continents (e.g., North America and Europe) and within the same countries, depending on resource availability and specialist availability [1, 5]. Assessing referral patterns may help clarify the definition, scope, and core skills required for undergraduate and postgraduate programs within dental curricula worldwide [5, 7, 8]. Thus, the present study aimed to describe and analyse the characteristics of referral letters from dental and medical referrers to a Middle Eastern university oral medicine service based in Saudi Arabia.

## Materials and methods

### Study design and population

This was a retrospective analysis of electronic and handwritten referrals to the Oral Medicine (OM) service at the Dental University Hospital, King Saud University Medical City, Riyadh, Saudi Arabia. The OM service provides diagnosis and management for oral mucosal diseases and orofacial pain disorders. It serves as a national dental and oral health centre, with the highest number of certified OM and orofacial pain specialists in the country, serving the capital’s 8.2 million residents [9]. Each clinician sees at least three new or follow-up patients in a 3-hour session weekly, as well as supervising postgraduate and undergraduate trainees in other clinical sessions. Inclusion and exclusion criteria are shown in Table 1.

**Table 1:** The study’s inclusion and exclusion criteria, as well as its characteristics

Inclusion criteria	All referrals to oral medicine services from January 2015, when the oral medicine service was officially recognised within the hospital, to April 2023. This includes the referrals of patients who were referred for pain, limited mouth opening, jaw clenching and clicking, orofacial hard and soft tissue swellings, trauma, sleep apnoea, dry mouth, oral ulceration, temporomandibular disorders (TMD), bruxism, deviation, locked mouth and myofascial pain.
Exclusion criteria	Whereas those with unclear handwriting and lacking clinical and referrer data were excluded.

### Study characteristics

The collected data from all referrals were represented and managed in a password-protected file, in line with national and international data protection laws and the Declaration of Helsinki [10]. The collected data included these related to referrals (the speciality of referrer) and referee (demographics, medical history, medication use, reason for referral and involved anatomical sites).

### Data analysis and representations

Microsoft Excel [v2.92.1] was used for data representation and analysis. Descriptive statistics [frequencies with percentage (%) for categorical variables, and the mean (± SD) and median for numerical variables collected] were used for the referrer characteristics and those related to patients’ demographics and clinical characteristics.

## Results

A total of 500 referrals, which represented all patients referred between 2015 and 2023, were initially reviewed for inclusion in the present analysis. After removing 23% of these records due to unclear referral letter handwriting, 5% lacking essential clinical data and 1.6% with broken papers were also excluded. Therefore, the remaining 350 records were considered in the present analysis. Of which, 62% were for females and 23% were males. The remaining 14% did not indicate the gender. The mean age of the referred patients was 36 years (± 16.6).

**Fig. 1:**
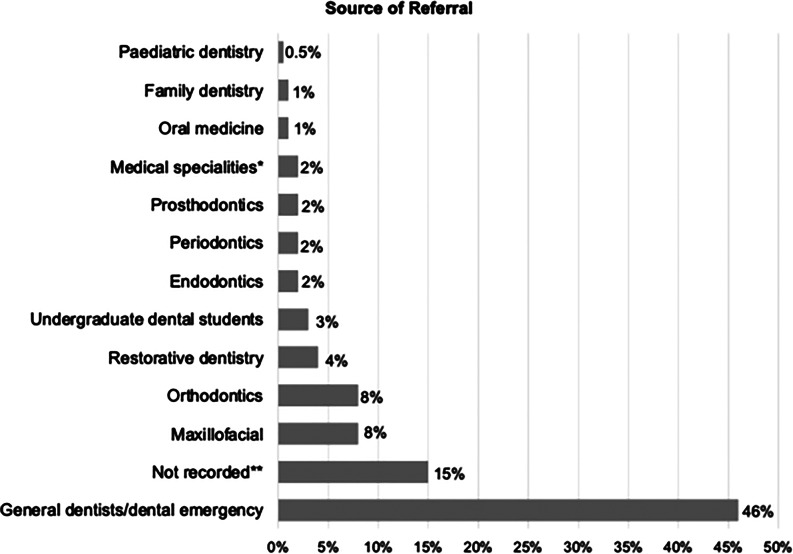
The source of referral to oral medicine services (*n* = 350). ^*^ENT, dermatology and internal medicine, ^**^The speciality of the referrer was not mentioned in the letter

The majority of referrals came from general dentists and dental emergency clinics accounting for 47% of the total records (Fig. 1).

Regarding the medical information in the reviewed referrals, 165 (47%) of the 350 records did not mention the relevant medical history, 100 (28%) noted no medical issues, 31 (8.8%) with diabetes mellitus, 30 (8.5%) with hypertension and 18 (5%) with diabetes mellitus and 13 (3%) with hypo or hyperthyroidism and 4 (1%) with multiple sclerosis. Other conditions, such as asthma, anaemia, rheumatoid arthritis and osteopenia, ear infection, dry eyes and mouth, sacral giant cell, eczema, fibromyalgia, epilepsy, depression, thalassemia, were noted in smaller percentages.

Furthermore, most of the 350 records (85%) lacked information about medication use. In contrast, the remaining 5 and 48 referrals noted no medication use or use of medications for diabetes, hypertension, asthma, antifungals, anticonvulsants, blood thinners, and osteoporosis medications.

With most referrals, one or more reasons were included; 50% and 45% noted pain and TMD, respectively. The TMD-related patient concerns included clicking (*n* = 69), limited mouth opening (*n* = 24), mandibular jaw deviation (*n* = 21), parafunctional activity (*n* = 21), locked jaw (*n* = 9) or not specified (*n* = 13). Furthermore, 12% of the referrers did not specify any reasons for referral (Table 2).

**Table 2:** The reasons for referral in the reviewed records (*n* = 350)

Reason for referral^*^	Number of subjects (%)
Pain^**^	176 (50.2%)
TMD and its related complaints	157 (44.8%)
Others^***^	38 (14%)
Ulceration	14 (4%)
Swelling	12 (3.4%)
Lesion	12 (3.4%)
Teeth attrition and sensitivity	4 (1%)
Mass	2 (0.5%)
Trauma	2 (0.5%)
Follow up	2 (0.5%)
Dry mouth	2 (0.5%)
Jaw radiolucency	2 (0.5%)
Obstructive sleep apnoea	1 (0.2%)
Not recorded	45 (12%)

^*^ Some records included more than two reasons for referral, ^**^ This includes oral mucosal pain and orofacial pain, ^***^ Others including reasons such as cheek biting, tooth wear, tinnitus, involuntary movement, oral candidiasis, reviewing lab results, tissue hardening, persistent headache, supernumerary teeth, jaw deviation, jaw hypermobility, teeth discolouration, deep bite and heart palpitations

Regarding the anatomical sites, no data were found among 55% (*n* = 194) of the reviewed records. Among the remaining 45%, 80 referrals pertained to the TMD, 17 to the tongue, and 15 to the buccal and labial mucosa. Other locations include the palate, teeth, lymph nodes, jaw, muscle, and lip, each with varying small percentages (Table 3).

**Table 3:** Anatomical sites for all reviewed records (*n* = 350)

Anatomical site^*^	Number of subjects (%)
Temporomandibular joint	80 (22.8%)
Others^**^	18 (5.1%)
Tongue	17 (4.8%)
Buccal/labial mucosa	15 (4.2%)
Palate	8 (2.2%)
Muscles of mastication	8 (2.2%)
Lip	6 (1.7%)
Teeth	5 (1.4%)
Jaw	5 (1.4%)
Not recorded^***^	195 (56.8%)

^*^Some records included more than two anatomical sites, ^**^Others included the gingiva, cervical lymph nodes, mucogingival junction, zygomaticomaxillary complex, retromolar trigone, inferior alveolar nerve, ear, salivary glands and floor of the mouth, ^***^The referral did not include the anatomical site

Furthermore, almost all referrals lack essential information, such as lesion description (e.g., colour and texture), clinical images, and provisional diagnoses. Thus, they were not included in the analysed data.

## Discussion

The present study presents significant data on the characteristics of referrals to OM in a Middle Eastern university-based clinical service, which were not previously detailed. This may help inform healthcare policymakers to adopt strategies that reduce diagnostic delays and redundant, unnecessary diagnostic procedures, often associated with a significantly increased oral disease burden and a worse quality of life [11]. Although this was a single-centre study, the findings likely reflect national and regional OM services, given the university hospital’s status as the region’s largest tertiary care facility, accessible to all residents and visitors from similar demographic and socioeconomic backgrounds across the country [12].

The findings of general dentists and dental emergency services, acting as primary referral sources, were consistent with those of other studies on referral records [5, 11, 13]. Although the number of referrals from physicians and medical specialists was considerable among OM practices in Australia [8], Brazil [14], Italy [11] and the USA [4, 15], it was notably low at present (< 5%). Such differences likely reflect factors related to global health care systems and their policies (e.g. referral pathway, access and eligibility for clinical care, insurance, funding and reimbursement), oral disease (e.g. prevalence and incidence in a population and disease burden) and clinicians (e.g. experience, clinical scope and awareness of the speciality) [2, 16–18]. It was also notable that some of the referrers were aligned with family dentistry, which is a recognised speciality in some countries, including Saudi Arabia. Their patient care approaches, along with those of family medicine specialists, aimed primarily to deliver a holistic oral and general health clinical service in community-based practices [19]. These approaches might also be useful for early detection of oral diseases, especially among individuals with risky health behaviours such as tobacco use and excessive alcohol drinking.

The higher percentage of referrals for female patients compared to males (62% of 350 patients) was in line with similar studies, where females represented 60% or more of the analysed referrals to OM [1, 4, 11, 13, 15]. However, there is varied evidence available about the prevalence of oral mucosal lesions among males and females, with a tendency for males who use tobacco to present with these lesions [20, 21]. It is also notable that the common referral reasons presently reported, including oral soreness, orofacial pain, and dry mouth, were notably higher in postmenopausal women than in men of the same age group [22]. Older adults often present with lesions from trauma, systemic disease, medication, autoimmune diseases, and infections. These may also include potentially premalignant or malignant disorders [23]. The present results showed a possibly higher mean age compared to similar studies, which reported mean ages ranging from 47 to 59 years [1, 4, 11, 13]. In contrast, the mean age in our study was significantly younger, at 36 years. This is likely due to the high prevalence of TMD and related complaints in young adults rather than older adults [24].

Currently, oral lesions account for only 2% of all referrals. This may reflect low awareness toward oral mucosal changes among health care professionals – a phenomenon noted among multiple studies around the globe [25]. Saudi Arabia and other Middle East-based studies have also mirrored findings with many of the surveyed HCPs having never known OM speciality, thought of as a purely academic speciality without diagnostic services [2, 26]. Moreover, those affiliated with family medicine demonstrated the lowest rates of OM awareness, despite its high relevance to the speciality [3, 26]. It may also reflect limited competency among HCPs in referring patients with suspicious mucosal changes. Patients with such lesions, including leukoplakia and erythroplakia, might have been referred elsewhere and already may have seen multiple HCPs [2, 27]. This often causes significant diagnostic delays and oral cancer-related complications [28] – the present study cohort is unlikely to be an exception.

To address the abovementioned limitations, there is an essence to integrating OM into medical curricula and clinical rotations, providing continuous professional development for medical and allied health care professionals, and supporting translational and clinical research on the bidirectional relationship between oral and general health [3, 29]. Incorporating basic oral health knowledge into activities of professional societies (e.g. annual meetings, membership portals, and clinical practice guidelines) can also promote early recognition and timely referral [25, 26]. Large language models (LLMs) have shown diagnostic accuracy comparable to experts in detecting oral diseases like mucosal lesions [30]. Models such as ChatGPT-5 and Gemini, with visual and textual capabilities, can support diagnostic workflows and improve outcomes [32]. HCPs may therefore adopt them to identify high-risk lesions, provide differential diagnoses, grade disease severity, interpret morphological features, and assess clinical reasoning related to mucosal changes [30–32]. Although their applicability in triaging referrals, diagnostic accuracy, and their ability to red-flag suspicious lesions (e.g., oral potentially malignant disorders) or serious orofacial pain conditions (e.g., giant cell arteritis) requires further assessments. Continuous multi-centre validation is needed to address practical limitations, including referral and medical record issues, automation biases, hallucinations, and overinterpretation of data [30, 33].

The study’s limitations included not analysing essential data, such as the distance between the patient’s home and the OM service, the referral-to-consultation interval, the number of clinicians seen before referral, the types of investigations or diagnostic tests, and the accuracy of initial versus final diagnoses [1, 4]. The analysis does not include inference about the study variables because the sample size is relatively small and the data are heterogeneous. For instance, it is unclear whether a patient’s reported pain is due to oral mucosal soreness, TMD-related pain, or neuropathic pain. As in previous studies [14, 34], missing key information on the referred speciality, demographics, medical history, and referral reason suggests that referrals were general and of low quality, potentially introducing a risk of reporting and selection bias. The study also omitted waiting list length, care accessibility, and the consultation appointment process [17]. Thus, there is a need for formalised clinical practice guidelines. These should sufficiently address the referrer’s information (name, rank, postcode/address, contact details) and patient details (demographics, detailed clinical data including the medical history review, history of chief complaint, tobacco use and alcohol drinking, clinical findings, investigations, clinical impression and initial diagnosis, management, and urgency of referral).

The 2030 World Health Organization’s Global Oral Health Care Action Plan aims for a 10% reduction in oral diseases, especially among high-risk individuals [12, 35]. Concerning labial and oral cavity cancers, they will be those who use tobacco, drink alcohol and have low socioeconomic status. This could possibly be supported by using the non-invasive brush cytology screening for potentially malignant and malignant oral mucosal lesions by general dental and medical practitioners. Their high diagnostic accuracy has been shown, but further work is needed to standardise the sampling and interpretation techniques to support their clinical adoption [36]. Whether timely referral and specialist diagnosis for patients with these cancers would improve their disease-free survival and overall survival remains uncertain. Although it appears to contribute to a favourable disease prognosis [28, 37]. This can be associated with tailored referral-to-OM pathways as well as public health surveillance and prevention [28]. Future work may consider tracking the quality and accuracy of referrals after OM service visits as well as the patient outcomes across multi-centre OM services. It is also important to assess whether information technology-enabled referral templates and triage improve the process.

## Conclusions

The present study provides detailed information about the referral characteristics of oral medicine in a significant Middle Eastern university hospital, which has not been adequately addressed previously. While many findings are consistent with international reports, the relatively low number of referrals from medical practitioners highlights a potential gap in referral practices. Although many findings were consistent with other global studies, the low referral numbers from medical practitioners emphasise the need for regional and international clinical practice guidelines for oral medicine referrals. These guidelines would ensure that patients receive timely OM care, reduce the future burden on healthcare services by enabling early interventions for oral mucosal changes, support national clinical networks in triaging high-risk patients, and facilitate the integration of the OM speciality and services within Saudi Arabia, the Middle East, and internationally.

## Data Availability

The dataset supporting the conclusions of this article can be obtained from the corresponding author upon a reasonable request (AM Alsoghier, aalsoghier@ksu.edu.sa).

## Abbreviations

LLMs: Large language models
OM: Oral medicine
TMD: Temporomandibular joint disorders
